# A new human chromogranin A (CgA) immunoradiometric assay involving monoclonal antibodies raised against the unprocessed central domain (145-245)

**DOI:** 10.1038/sj.bjc.6690013

**Published:** 1999-09-24

**Authors:** F Degorce, Y Goumon, L Jacquemart, C Vidaud, L Bellanger, D Pons-Anicet, P Seguin, M H Metz-Boutigue, D Aunis

**Affiliations:** CIS biointernational, Division In Vitro Technologies, Bagnols-sur-Cèze, 30203, France; Institut National de la Santé et de la Recherche Médicale, INSERM U-338 Biologie de la Communication Cellulaire, Strasbourg, 67084, France

**Keywords:** chromogranin A, monoclonal antibodies, epitope mapping, proteolysis, immunoradiometric assa

## Abstract

Chromogranin A (CgA), a major protein of chromaffin granules, has been described as a potential marker for neuroendocrine tumours. Because of an extensive proteolysis which leads to a large heterogeneity of circulating fragments, its presence in blood has been assessed in most cases either by competitive immunoassays or with polyclonal antibodies. In the present study, 24 monoclonal antibodies were raised against native or recombinant human CgA. Their mapping with proteolytic peptides showed that they defined eight distinct epitopic groups which spanned two-thirds of the C-terminal part of human CgA. All monoclonal antibodies were tested by pair and compared with a reference radioimmunoassay (RIA) involving CGS06, one of the monoclonal antibodies against the 198–245 sequence. It appears that CgA C-terminal end seems to be highly affected by proteolysis and the association of C-terminal and median-part monoclonal antibodies is inadequate for total CgA assessment. Our new immunoradiometric assay involves two monoclonal antibodies, whose contiguous epitopes lie within the median 145–245 sequence. This assay allows a sensitive detection of total human CgA and correlates well with RIA because dibasic cleavage sites present in the central domain do not seem to be affected by degradation. It has been proved to be efficient in measuring CgA levels in patients with neuroendocrine tumours. © 1999 Cancer Research Campaign


*British Journal of Cancer* (1999) **79**(1), 65–71
© 1999 Cancer Research Campaign

### A new human chromogranin A (CgA) immunoradiometric assay involving monoclonal antibodies raised against the unprocessed central domain (145–245)

**F Degorce[1], Y Goumon[2], L Jacquemart[1], C Vidaud[1], L Bellanger[1], D Pons-Anicet[1], P Seguin[1], MH Metz-Boutigue[2] and D Aunis[2]**

[1]CIS biointernational, Division In Vitro Technologies, BP175, 30203 Bagnols-sur-Cèze, France; [2]Institut National de la Santé et de la Recherche Médicale, INSERM U-338, Biologie de la Communication Cellulaire, 67084 Strasbourg, France

**Summary** Chromogranin A (CgA), a major protein of chromaffin granules, has been described as a potential marker for neuroendocrine tumours. Because of an extensive proteolysis which leads to a large heterogeneity of circulating fragments, its presence in blood has been assessed in most cases either by competitive immunoassays or with polyclonal antibodies. In the present study, 24 monoclonal antibodies were raised against native or recombinant human CgA. Their mapping with proteolytic peptides showed that they defined eight distinct epitopic groups which spanned two-thirds of the C-terminal part of human CgA. All monoclonal antibodies were tested by pair and compared with a reference radioimmunoassay (RIA) involving CGS06, one of the monoclonal antibodies against the 198–245 sequence. It appears that CgA C-terminal end seems to be highly affected by proteolysis and the association of C-terminal and median-part monoclonal antibodies is inadequate for total CgA assessment. Our new immunoradiometric assay involves two monoclonal antibodies, whose contiguous epitopes lie within the median 145–245 sequence. This assay allows a sensitive detection of total human CgA and correlates well with RIA because dibasic cleavage sites present in the central domain do not seem to be affected by degradation. It has been proved to be efficient in measuring CgA levels in patients with neuroendocrine tumours.

**Keywords:** chromogranin A; monoclonal antibodies; epitope mapping; proteolysis; immunoradiometric assay

Human chromogranin A (hCgA) is a 48-kDa protein with a pI of 4.9, encompassing 439 amino acids (Konecki et al, 1987). It belongs to the granin family with which it shares numerous structural and physiological similarities (Simon and Aunis, 1989 for a review). CgA is largely distributed in secretory granules of endocrine and neuroendocrine cells and constitutes, with other members of the granin family, one of their most abundant components (Cetin and Grube, 1991).

It has been shown that CgA plays an essential prohormone role through the release of bioactive peptides produced by its intragranular and extracellular proteolysis of the protein (Barbosa et al, 1991; Metz-Boutigue et al, 1993). Many studies have described natural peptides in human, as well as in bovine samples, including pancreastatin, vasostatins/β-granin, chromostatin, WE-14, parastatin/GE-25 (Iacangelo and Eiden, 1995 for a review) and prochromacin (Strub et al, 1996). These peptides display several biological properties, including antibacterial activity as recently demonstrated by Strub et al (1996) for bovine prochromacin. The proteolysis concerns mainly the ten dibasic sites distributed along the hCgA sequence, although other types of cleavage have been described elsewhere on the sequence (Metz-Boutigue et al, 1993). It has also been shown that this process is recurrent for C- and N-terminal ends (Barbosa et al, 1991; Metz-Boutigue et al, 1993).

*Received 8 January 1998*
*Revised 3 April 1998*
*Accepted 7 April 1998*

*Correspondence to:* F Degorce

Furthermore, several studies demonstrated that CgA could be degraded in a tissue-specific manner, which led to important disparities in the tissular distribution of the peptides released (Cetin and Grube, 1991; Curry et al, 1991; Watkinson et al, 1991). It is likely that the proteolysis properties may be responsible for the variability of the fragments found in normal and pathological tissues, blood or urine. Corti et al (1996*a*) recently confirmed this diversity by demonstrating the occurrence of different antigenic profiles among a population of patients affected with phaeochromocytoma. Apart from its differential expression in normal and neoplastic tissues, many studies have demonstrated the diagnostic value of hCgA measurement in blood. Levels of total circulating hCgA are significantly elevated in phaeochromocytomas, carcinoid and pancreatic endocrine tumours (O’Connor and Deftos, 1986). These data have been confirmed and extended to other neoplasia: neuroblastoma and gastrointestinal tumours (Eriksson et al, 1990; Hsiao et al, 1990; Stridsberg et al, 1995). Other authors have also described how the occurrence of plasma hCgA in prostatic carcinoma may be the sign of an unfavourable evolution (Cussenot et al, 1996; Deftos et al, 1996).

Circulating CgA is usually determined by competition assays, similar to the immunoassay described by O’Connor and Bernstein (1984), either with radiolabelled or with enzyme-conjugated CgA (Dillen et al, 1989). More recently, sandwich methods involving monoclonal or polyclonal antibodies have been published (Bender et al, 1992; Syversen et al, 1994; Corti et al, 1996*a*).

The proteolysis of CgA and the multiplicity of circulating fragments suppose an immunoassay configuration able to detect most, if not all, of these entities. The aim of the present study was to develop a sandwich assay which would enable the measurement of intact and fragmented circulating human CgA. We assumed that a combination of monoclonal antibodies (mAbs) directed against the median part of the protein, presumably less exposed to proteolysis because of post-translational modifications (Strub et al, 1997), would satisfy such requirements. We therefore generated and selected 24 mAbs against hCgA (human native chromogranin A) and rhCgA (recombinant human chromogranin A). Epitope mapping using both Biacore and hCgA proteolytic peptides was carried out to specifically address the different epitopes to the CgA protein. Screening of the different mAb pairs against normal and pathological sera led us to select a sandwich assay, which did not appear to be affected by hCgA proteolysis because the two mAbs involved possess contiguous epitopes in the central part of hCgA. Finally, this new assay has been proved to be efficient in measuring CgA levels in patients with neuroendocrine tumours.

### MATERIALS AND METHODS

### Purification of CgAs

Human adrenal phaeochromocytoma was used to purify hCgA according to the method described previously by O'Connor et al (1984). Final protein separation was carried out by 2D-gel electrophoresis according to O'Farrell's original technique (O'Farrell, 1975) modified by Bader and Aunis (1983). CgA was then electroeluted from gels (Metz-Boutigue et al, 1993). *Escherichia coli* BL21 (DE3) strain expressing rhCgA was grown as described elsewhere (Taupenot et al, 1995). Production of rhCgA was carried out according to the method described by the same authors.

### Radioiodination of purified CgA

Purified native or recombinant CgAs were radiolabelled with $^{125}$I-labelled sodium iodide according to the chloramine T method (Hunter and Greenwood, 1962) at a specific activity of 1500 kBq $\mu g^{-1}$. Free iodine was removed by gel filtration on a Sephadex-G25 gel (Pharmacia Biotech, Orsay, France). Radiolabelled protein fractions were pooled and diluted in phosphate-buffered saline (PBS) pH 7.2 containing 45 mmol $l^{-1}$ sodium azide and 0.5% bovine serum albumin (BSA).

### Immunizations

Balb/c mice, 6–8 weeks old, were respectively immunized intraperitoneally with 10 µg of purified hCgA or rhCgA in Freund's complete adjuvant. The following intraperitoneal doses were made in Freund's incomplete adjuvant at 1-month intervals. Immunizations of mice were monitored by serum titration via immunoprecipitation of [$^{125}$I]hCgA. All animals were handled according to French law.

### Production and screening of hybridomas

The mice were boosted intravenously with 10 µg of hCgA in 0.9% sodium chloride solution 3 days before cell fusion. Spleen cells were isolated and fused with myeloma P3-X63 Ag8.653 cell line in the presence of 37% polyethylene glycol (MW 1540). Positive clones were identified by immunoprecipitation of [$^{125}$I]hCgA. mAbs from positive clones were produced from mouse ascites and purified using protein A–Sepharose (Pharmacia Biotech).

### Mapping of monoclonal antibodies

The different families of CgA epitopes were identified by testing the possible combinations of mAbs on the Biacore (Biacore, Saint Quentin-en-Yvelines, France) according to the manufacturer's instructions. Biacore is a system for realtime biomolecular interaction analysis based on surface plasmon resonance. Briefly, the sensorchips were prepared by coupling anti-mouse antibodies (CIS biointernational, Bagnols-sur-Cèze, France) with *N*-hydroxysuccinimide and water-soluble carbodiimide before sequential injection of the following reagents in 200 µl of HEPES buffered saline 50 mM, EDTA 15 mM, sodium chloride 0.75 M, pH 7.4: (i) solid phase antibody, (ii) rhCgA at 20 µg $ml^{-1}$, (iii) mixture of normal mouse immunoglobulins for saturation at 200 µg $ml^{-1}$ and (iv) detecting antibody. All mAbs were injected at 50 µg $ml^{-1}$. The sensorchip was regenerated with 0.1 mmol $l^{-1}$ hydrochloric acid. Signals expressed in resonance units (RU) allowed us to work out epitope compatibilities and the performance of the different pairs.

### Mapping with rhCgA-derived peptides

Recombinant hCgA (2 nmol) was digested for 2 h at 37°C with endoproteinase Lys-C or trypsin at a protein/proteinase ratio of 1000:1 in 100 mmol $l^{-1}$ Tris-HCl, pH 8.3. Generated rhCgA-derived peptides were then separated on a Macherey Nagel 300-5C18 column (250 mm × 4 mm). Absorbance was monitored at 214 nm, and the solvent system consisted of 0.1% trifluoroacetic acid in water (solvent A) and 0.1% trifluoroacetic acid in acetonitrile (solvent B). Material was eluted at a flow rate of 0.7 ml $min^{-1}$ using successive gradients of solvent B in A of 0–25% for 10 min and of 25–75% for 50 min. Each peak fraction was collected and concentrated by evaporation, but not to complete dryness.

Aliquots of digested rhCgA high-performance liquid chromatography (HPLC) fractions were dotted on nitrocellulose sheets. Membranes were quickly washed with sodium chloride/inorganic phosphate (25 mM sodium phosphate pH 7.5 containing 0.9% sodium chloride) and incubated for 2 h at room temperature with mAbs diluted 1:1000 in sodium chloride/inorganic phosphate. The second antibody was an anti-mouse IgG conjugated to alkaline phosphatase (Biorad, Ivry-sur-Seine, France). Enzymatic reaction took place in 100 mmol $l^{-1}$ Tris-HCl pH 8.5, 100 mmol $l^{-1}$ sodium chloride, 50 mmol $l^{-1}$ magnesium chloride containing 0.4 mmol $l^{-1}$ nitro-blue tetrazolium and 0.38 mmol $l^{-1}$ 5-bromo-4-chloro-3-indolyl phosphate (Boehringer Mannheim, Meylan, France).

The sequence of purified peptides was determined by automatic Edman degradation on an Applied Biosystems 473A microsequencer. Samples purified by HPLC were loaded on polybrene-treated and precycled glass-fibre filters (Metz-Boutigue et al, 1993). Phenylthiohydantoin amino acids were identified by chromatography on a C-18 column (PTH C-18, 2.1 mm × 200 mm). Mass spectra analysis (Maldi-Tof) was obtained according to the procedure previously reported (Goumon et al, 1996).

### Reference radioimmunoassay for hCgA

mAb CGS06 against hCgA was used to design this RIA (radioimmunoassay). Purified rhCgA used for the calibrators was diluted in normal human serum. The calibrator concentrations ranged from 0 up to 1600 ng $ml^{-1}$. After combining 50 µl of calibrator or sample to be assayed, 300 µl of radiolabelled hCgA

 © Cancer Research Campaign 1999

**Table 1** Characteristics of mAbs obtained. Names in bold type indicate antibodies raised against native hCgA. Epitope mapping as well as stoichiometry which is expressed as mole of rhCgA/mole of mAb were carried out with Biacore. mAb CGS03 stoichiometry could not be tested (n.t.)

| Name | Isotype | Stoichiometry | Epitope group |
|---|---|---|---|
| **CGS01** | IgG1 | 0.431 | 1 |
| **CGS03** | IgG1 | n.t. | |
| **CGS04** | IgG2b | 0.365 | |
| **CGS12** | IgG1 | 0.364 | |
| **CGS06** | IgG2a | 0.503 | 2 |
| **CGS08** | IgG1 | 0.457 | |
| **CGS10** | IgG1 | 0.182 | 3a |
| **CGS05** | IgG1 | 0.157 | |
| CGS23 | IgG2a | 0.604 | |
| CGS24 | IgG2a | 0.521 | |
| CGS25 | IgG2a | 0.553 | |
| CGS26 | IgG2a | 0.356 | |
| CGS29 | IgG1 | 0.621 | 3b |
| CGS34 | IgG1 | 0.536 | |
| CGS35 | IgG1 | 0.539 | 3c |
| CGS30 | IgG2a | 0.327 | 4 |
| CGS31 | IgG1 | 0.198 | |
| CGS33 | IgG1 | 0.383 | |
| CGS36 | IgG1 | 0.184 | |
| CGS17 | IgG1 | 0.162 | 5 |
| CGS19 | IgG1 | 0.361 | |
| CGS20 | IgG1 | 0.254 | 6 |
| CGS21 | IgG1 | 0.183 | 7 |
| CGS32 | IgG1 | 0.262 | 8 |

(2 kBq per tube) and 150 µl of purified CGS06 mAb solution (80 ng ml$^{-1}$) in a tube, the first incubation took place for 24 h at room temperature under gentle shaking. Bound complexes were separated for 20 min at room temperature by adding 75 µl of sheep anti-mouse immunoglobulins diluted to 1:10 in PBS and 50 µl of human normal serum. One millilitre of 6% polyethylene glycol (MW 6000) was added and pellets were counted on a γ-counter (Crystal II, Packard Instrument, Rungis, France) after centrifuging at 2000 ***g*** for 15 min (961R, Seroa, Monaco).

### Selection of a suitable pair of mAbs for immunoradiometric assays (IRMA)

mAbs whose epitopes had been mapped to hCgA were used for the screening of an optimal antibody combination for the measurement of CgA. All pairs were tested against two pathological plasma pools from several patient samples with phaeochromocytoma (PP) and carcinoids (CP) previously assayed in the RIA, as well as against a pool of plasmas coming from healthy individuals (NP).

Three combinations that involved epitopes spanning the C-terminal end of the protein (CGS29 and CGS04) were retained for further testing on a larger population of normal subjects ($n$ = 20) and of 39 patients suffering from neuroendocrine tumours: phaeochromocytoma, bronchial and intestinal carcinoids, pancreatic endocrine neoplasia, medullary thyroid carcinoma (MTC). All plasmas were obtained and used in accordance with local ethical rules.

### *Experimental procedure*

All antibodies were brought to a concentration of 10 µg ml$^{-1}$ in PBS pH 7.5 and coated to polystyrene tubes (Greiner France, Poitiers, France). Concurrently, mAbs were radiolabelled with $^{125}$I-labelled sodium iodide using the chloramine T method as described for rhCgA, at a specific activity of 400 kBq µg$^{-1}$. Calibrators were made as described earlier for RIA. Assay buffer consisted of disodium hydrogen phosphate/potassium dihydrogen phosphate 50 mmol l$^{-1}$, EDTA 1 mmol l$^{-1}$, sodium azide 15 mmol l$^{-1}$, BSA 1%, pH 7.0. Briefly, 1 ml of assay buffer and 50 µl of standard or sample were mixed in coated tubes. Incubation took place for 18 h at room temperature. Tubes were washed twice with 2 ml of a solution of 0.3% Tween 20. One millilitre of radiolabelled mAb was then added to each tube and incubated for 2 h at room temperature under gentle agitation. Tubes were washed again as previously described and radioactivity was counted on the γ-counter.

### RESULTS

### Purification of CgA

After microsequencing, the degree of purity of CgA preparations was checked by 2D-gel electrophoresis as described by Metz-Boutigue et al (1993). The two-dimensional gel showed a major spot (70 kDa; pI 5–5.5) corresponding to intact CgA and minor CgA-derived fragments, the latter representing 5% of total protein. This pattern of the gel was consistent with results obtained by Taupenot et al (1995).

### Generation of monoclonal antibodies

Two generations of antibodies were obtained with hCgA and rhCgA respectively (Table 1). Eleven cell fusions were carried out with mice immunized with hCgA, producing eight hybridomas. The two cell fusions with animals immunized against the recombinant protein generated 16 different clones. A total of 17 IgG1, six IgG2a and one IgG2b were produced for further characterization.

### Biacore mapping

All possible pairs of mAbs were tested with Biacore using rhCgA. The 24 mAbs could be distributed into eight distinct groups (Table 1). mAbs from group 3, however, could be further subdivided because of partial inhibitions existing between CGS10-like antibodies and CGS29, CGS34 and CGS35.

Stoichiometry was determined for each mAb by calculation of the ratio RU (resonance units) of bound rhCgA/RU of immobilized mAb. Values were found to range from 0.157 to 0.621 mole rhCgA/mole mAb. No obvious relationship could be established between the type of immunogen used and the stoichiometry, except for mAbs from group 3a. In this group, the stoichiometry of CGS10 and CGS05 raised against human native CgA is significantly weaker than that of second generation mAbs against the recombinant protein.

One mAb of each main group was retained for further characterization: CGS04, CGS06, CGS10, CGS17, CGS20, CGS21, CGS30 and CGS32 were selected. In spite of a weaker stoichiometry, CGS10 and CGS17 were also included in this series because of better hybridoma productivity.

A

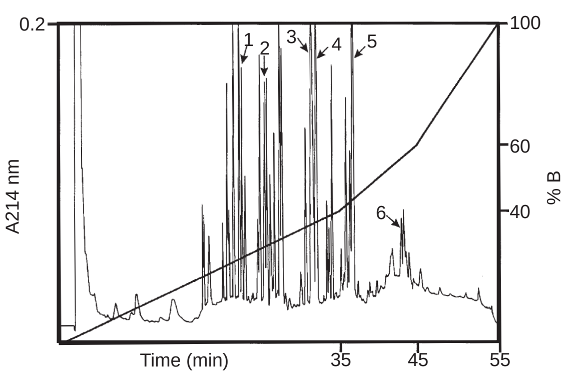

B

| Fraction | Antibody | N-terminal sequence | Mass (Da) | Fragment |
|---|---|---|---|---|
| 1 | CGS17 | RLEGQEE | 2053.9 | 339–355 |
| 2 | CGS30 | AEGNNQA | 5635.8 | 145–197 |
| 3 | CGS20 | EIRKGESRS | 6224.8 | 246–303 |
| 4 | CGS06 | GLSAEPG | 5265.4 | 198–245 |
| 5 | CGS21 | LXFRARA | 5145.8 | 356–400 |
| 6 | CGS32 | GXTEVMKXIV | 43532 | 10–400 |
| * | CGS04 | LEGQEE | n.d. | 340–394 |
| * | CGS10 | GYPEEK | n.d. | 395–439 |

**Figure 1** (**A**) HPLC profile of digested rhCgA with endoprotease Lys-C. (**B**) Characteristics of fragments recognized by the different mAbs. mAbs CGS04 and CGS10 were tested by Western blot and the corresponding immunodetected spot was sequenced according to Metz-Boutigue et al (1993). n.d., mass spectroscopy was not carried out for these mAbs; X, non-identified residue. Location of CgA-derived fragments was obtained according to CgA sequence (Koneki et al, 1987)

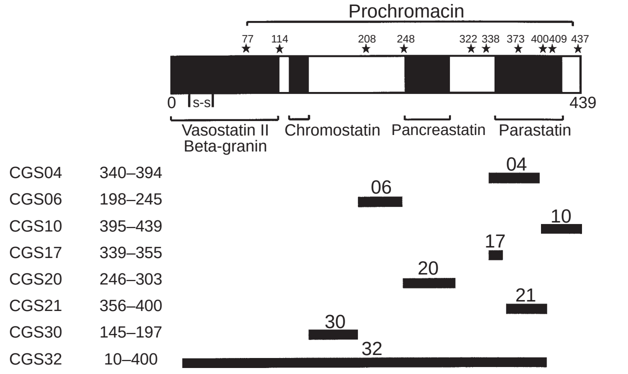

**Figure 2** Human CgA sequence and position of the different epitopes. Stars and corresponding numbers indicate the dibasic cleavage sites. mAb CGS32 epitope could not be identified precisely (10–400)

### Mapping of monoclonal antibodies after endoproteinase Lys-C digestion of rhCgA

Recombinant hCgA was digested with endoproteinase Lys-C and all the mAbs mentioned above were tested against each HPLC aliquot fraction of digested rhCgA (Figure 1). mAbs CGS17 and CGS21 recognized the C-terminal domain of the protein with a high specificity for regions 339–355 and 356–400 respectively (Figure 2). In addition, mAbs CGS04 and CGS10 were directly tested by Western blot, and the immunodetected fragments were in the C-terminal region of CgA (340–394 and 395–439). mAbs CGS06, CGS20 and CGS30 were specific for the median part of the protein core 198–245, 246–303 and 145–197 (Figure 2). Finally, mAb CGS32 was strongly reactive with a large fragment 10–400; this antibody immunodetected a central fragment corresponding to sequence 198–245, but reaction was weak.

### Selection of a suitable mAb pair

The two pathological pools (PP and CP) were made from several EDTA plasmas coming respectively from patients with phaeochromocytoma and intestinal carcinoids. CgA levels, as determined by the reference RIA, were found to be 675 ng $ml^{-1}$ for PP and 1600 ng $ml^{-1}$ for CP. These values were compared with that of the normal pool which was determined to be 40 ng $ml^{-1}$ by the reference RIA.

The results for the 56 mAb combinations tested (Table 2) show the values obtained for the highest rhCgA calibrator at 1200 ng $ml^{-1}$, as well as the concentration ratios for PP/NP and CP/NP. For 26 combinations, CP was out of range and had to be further diluted. This was also the case for PP in three combinations.

Combinations using CGS21 as a tracer hardly detect rhCgA, as shown by a bound/total ratio of less than 5% at the 1200 ng $ml^{-1}$ calibrator. This was also the case, to a minor extent, for tracer CGS10 (three combinations out of seven and low B/T in general). All other systems were able to detect rhCgA and showed a significant rise in the signal with increasing rhCgA concentrations.

Concerning the detection of CgA in pathological pools, several types of combinations emerge. First, several systems gave a negative or a poor discrimination of the pools, i.e. CgA level measured in PP was never greater than twice that of NP. This situation applied to most of the combinations involving CGS10 and CGS04, either immobilized or used as tracers. Systems involving CGS17 or CGS20 gave a weaker discrimination, especially when associated with antibodies against the 340–439 region of the protein (CGS17, CGS21, CGS04 and CGS10). This was particularly true when the two mAbs were used as capture antibodies.

A second behaviour could be identified through systems such as CGS21/CGS06*, CGS21/CGS30* or CGS32/CGS06* which discriminated the two pathological pools positively but did not differentiate them from each other.

Finally, 19 combinations showed PP/NP ratios of greater than 2 as well as high CP/NP ratios, with substantially elevated CgA levels in CP italic data in Table 2). When the combination of CGS21/CGS32* is excluded (i.e., the system could not be reversed), it seems that only heterologous combinations involving mAbs CGS06, CGS30 and CGS32 were able to measure increasing CgA concentrations in NP, PP and CP with results close to the reference RIA. All combinations selected involved epitopes mapped to the median part of the protein.

To further check these conclusions, three different systems using tracer mAbs spanning the C-terminal domain of the protein were tested on a larger population of samples (Figure 3) and compared with the reference RIA. Because mAb CGS10 behaved poorly as a tracer when associated with CGS06, mAb CGS29, which shares the same epitope as CGS10, was used for this experiment.

Analysis of the pathological/normal ratios at the 95th percentile by Wilcoxon matched-pairs signed-ranks showed that

© Cancer Research Campaign 1999

**Table 2** Evaluation of the different mAb combinations on normal vs pathological plasma pools

| Solid phases | Tracers | | | | | | | |
|---|---|---|---|---|---|---|---|---|
| | **CGS32** | **CGS30** | **CGS06** | **CGS20** | **CGS17** | **CGS21** | **CGS04** | **CGS10** |
| CGS32 | | ***48.13***[a]<br>*16.4*[b]<br>*23.3*[c] | **30.89**<br>24.3<br>28.1 | ***7.01***<br>*8.8*<br>*50.3* | ***15.63***<br>*4.3*<br>*28.0* | **3.01**<br>18.0<br>39.6 | **32.17**<br>1.9<br>15.6 | **3.53**<br>1.0<br>9.3 |
| CGS30 | ***21.56***<br>*10.9*<br>*17.7* | | ***57.56***<br>*16.0*<br>*25.0* | ***22.04***<br>*5.6*<br>*21.8* | ***29.2***<br>*3.0*<br>*29.2* | **3.38**<br>14.4<br>31.3 | **60.57**<br>1.2<br>9.3 | **9.40**<br>0.5<br>7.5 |
| CGS06 | ***18.8***<br>*11.3*<br>*18.5* | ***65.51***<br>*9.9*<br>*18.2* | | ***12.8***<br>*4.0*<br>*23.2* | **20.38**<br>1.6<br>22.0 | **2.6**<br>8.0<br>18.5 | **45.17**<br>1.0<br>10.6 | **3.92**<br>0.9<br>3.8 |
| CGS20 | **15.11**<br>1.3<br>10.2 | ***43.22***<br>*2.6*<br>*12.8* | ***25.43***<br>*2.0*<br>*15.6* | | **8.29**<br>1.7<br>26.6 | **1.42**<br>2.5<br>11.3 | **30.70**<br>0.8<br>7.6 | **1.61**<br>0.0<br>0.9 |
| CGS17 | ***17.67***<br>*2.3*<br>*14.2* | ***62.66***<br>*2.4*<br>*22.4* | ***49.52***<br>*2.3*<br>*24.0* | ***21.29***<br>*2.0*<br>*20.9* | | **2.7**<br>2.4<br>20.7 | **60.86**<br>1.0<br>10.4 | **7.81**<br>0.5<br>5.4 |
| CGS21 | ***15.86***<br>*9.6*<br>*20.4* | **67.46**<br>13.0<br>14.6 | **50.52**<br>16.2<br>16.2 | ***15.4***<br>*5.5*<br>*11.7* | ***27.53***<br>*3.3*<br>*19.4* | | **48.57**<br>1.1<br>5.5 | **7.82**<br>0.0<br>0.7 |
| CGS04 | **28.83**<br>1.1<br>4.7 | **76.53**<br>1.2<br>13.5 | **55.53**<br>1.1<br>28.2 | **26.71**<br>1.1<br>24.3 | **55.05**<br>0.9<br>17.3 | **3.62**<br>1.2<br>14.3 | | **19.57**<br>0.7<br>16.4 |
| CGS10 | **12.39**<br>0.1<br>3.9 | **33.96**<br>0.7<br>5.0 | **17.49**<br>0.8<br>9.0 | **7.77**<br>0.5<br>6.1 | **11.65**<br>0.5<br>4.0 | **1.44**<br>0.9<br>4.0 | **30.54**<br>0.6<br>4.3 | |

[a]Per cent binding at 1200 ng $ml^{-1}$ of rhCgA. [b] and [c]CgA concentration ratios PP/NP and CP/NP respectively. Dark grey squares indicate low-sensitivity systems. Light grey squares stand for negative or poor discriminative systems. White squares with underlined italic type show combinations that discriminate PP and CP positively as well as one from the other. Ratios of PP/NP and CP/NP were found to be 16.9 and 40 in the reference RIA.

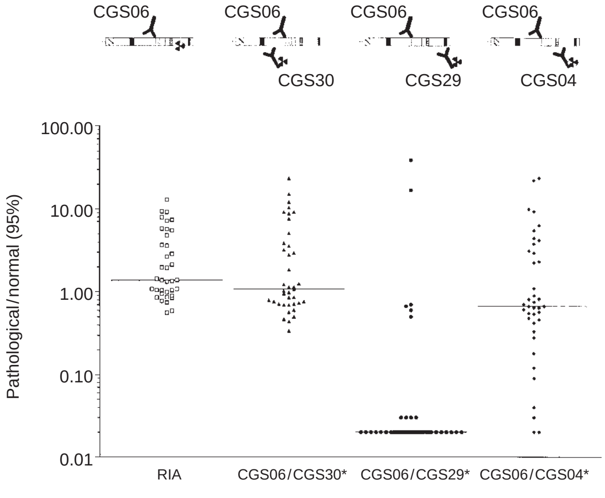

**Figure 3** Comparison of three different immunoradiometric assays vs the reference RIA. Data are expressed as the concentration ratio of pathological plasma/95th percentile value of normal population ($n$ = 20). Line segments indicate the median value for each group

CGS06/CGS30* and the reference RIA, as well as CGS06/CGS04* and the RIA, are closely related ($P < 0.0001$ and $P = 0.011$ respectively). In contrast, CGS06/CGS29* is significantly different from the RIA ($P = 0.738$). The CGS06/CGS30* combination with two contiguous epitopes within the median part of CgA gave a very similar distribution of the samples when compared with the RIA. Normal values ranged from 33 to 94 ng $ml^{-1}$ and the cut-off value was fixed at 86 ng $ml^{-1}$ (95th percentile of normal population). However, the use of CGS29 tracer, whose epitope is CGS10-like, led to a dramatic decrease in the concentrations measured. The rhCgA calibrator at 1200 ng $ml^{-1}$ was detected at a significant level (20% of B/T). However, concentrations measured for normal plasmas ranged from 0 to 55 ng $ml^{-1}$ and CgA level was undetectable in 50% of the population. With this system, only two samples could be measured above the cut-off (30 ng $ml^{-1}$) and they both came from patients with intestinal carcinoids. Furthermore, 29 samples out of 39 had an undetectable level of CgA, irrespective of their origin.

When an intermediate mAb such as CGS04 was used, the dispersion was improved although the concentrations of 8 samples out of 39 were clearly diminished, including two samples with no CgA. All samples from healthy individuals could be detected (range 15–71 ng $ml^{-1}$) and enabled us to fix the cut-off value at 67 ng $ml^{-1}$. All these results clearly show that CGS06/CGS30* is the closest combination to the reference RIA.

### DISCUSSION

In the present study, 24 monoclonal antibodies were generated against native and recombinant human CgA, and we report here their characterization and the development of a sandwich IRMA for hCgA.

© Cancer Research Campaign 1999 

The epitope analysis carried out raises several questions. The experiment for the selection of a mAb pair showed that CGS04 perfectly detected rhCgA contained in calibrators, but failed to recognize hCgA in PP when associated to any other mAb and irrespective of its position in the system. Similarly to CGS10, which shows the same behaviour, it appears, therefore, that the proteolysis recurrently affecting both ends of the protein (Gill et al, 1992; Metz-Boutigue et al, 1993) is particularly active against the last three, perhaps four, cleavage sites (373, 400, 409 and 437). It is important to stress that the processing is complex and governed by many different aspects. Among those, Strub et al (1997) showed that proteolysis of bovine CgA could depend on post-translational modifications. For instance, the putative cleavage site 373–374 may or may not constitute a proteolytic target, depending on the phosphorylation of serine-380. In contrast, CGS21, whose epitope considerably overlaps that of CGS04, behaves quite differently, and its association with antibodies against the median part of hCgA enables the detection of PP. Given the length of the sequences deduced, it is most likely that the actual CGS04 epitope is located in the C-terminal end of the 340–394 region; while CGS21 recognizes the N-terminal part of the 356–400 sequence, the cleavage site 373–374 is lying in between. The nature of the CGS17 epitope remains unclear. Mapping with rhCgA peptides indicates a very restricted sequence (339–355), upstream of the CGS21 epitope. Surprisingly, CGS17 barely detects PP when associated with median-type antibodies. Cleavage sites located upstream do not seem to be involved either, as shown by a positive association of CGS21 with the same antibodies. Strub et al (1997) have described a phosphorylation site on serine-307 of bovine CgA that is conserved in hCgA (ser-315) and, thus, close to the CGS17 epitope. Differential phosphorylation of CgA in normal or pathological tissues – through the activation of this mechanism in tumours – may explain such a difference in terms of immunoreactivity because the rhCgA against which CGS17 was raised is not post-translationally modified.

Median-type mAb epitopes (CGS20, CGS06, CGS30) are likely to recognize the CgA core as well. The three mAbs detect native and recombinant hCgA equally well, although CGS20 and, above all, CGS06 are close to two putative *O*-glycosylation targets (Strub et al, 1997). Finally, CGS32 could not be addressed accurately. Given its pattern (i.e. discrimination of the different pools), it is probably directed against the median part of CgA. All these results contradict to a certain extent the antigenic profiles already described by Gill et al (1992) and Corti et al (1996*b*). It first appears that most of the mAbs we obtained are directed against regions of hCgA which were shown to be poorly antigenic by these authors, apart from the CGS04 and CGS06 epitope groups that match respectively the highly antigenic domains 375–394 and 222–230. Unexpectedly, none of the mAbs detected the N-terminal third of the protein. This result remains unexplained because both immunogens used contained hardly any truncated hCgA. However, this part of the protein is well preserved among different species and it may be less immunogenic.

As first published by O'Connor and Bernstein (1984), many CgA immunoassays involved radiolabelled CgA and polyclonal antibodies. In these configurations, CgA measurements reflect levels of intact as well as truncated CgA. To detect most of these entities, CGS06, against the 198–245 sequence of hCgA, was chosen to develop a reference RIA. Results obtained with this assay on a population of various neuroendocrine neoplasia were consistent with previously described competition assays (data not shown). Following the same assumption, we wanted to check whether the association of CGS06 with other mAbs against CgA could allow the assessment of total hCgA in a comparable manner. The evaluation of all possible mAb combinations with three plasma pools showed that only those systems involving mAbs against the median domain of hCgA effectively discriminate. We excluded all the combinations that detected rhCgA weakly because the concentrations calculated may be inaccurate. For instance, all combinations with the tracer CGS21 were eliminated. In these cases, minor contaminations of calibrators by smaller rhCgA fragments could be suspected, although they cannot have such an influence in the second step of the assay. Moreover, combinations with CGS04 as tracer, whose epitope is immediately downstream from that of CGS21, perfectly detect increasing concentrations of rhCgA. It is therefore very likely that radioiodination by chloramine T simply alters CGS21 immunoreactivity. CGS10 reactivity seems to be affected to a lesser extent (apart from in the CGS04/CGS10* system) for the same reasons. It must be stressed that this problem could be ruled out when CGS10 was replaced by CGS29, a mAb from the same group, for the last comparative test, which confirmed yet again the full integrity of the rhCgA calibrator.

Concerning the results obtained from the pathological pools, it is clear that a major degradation occurs at the C-terminal end of hCgA. Only combinations involving median-type mAbs against the large domain 145–303 allow a reliable discrimination especially when corresponding epitopes are contiguous, as for CGS06 and CGS30. Besides, these data confirm that the cleavage site 208 separating the two epitopes is relatively unaffected. The final test with systems associating immobilized CGS06 with other mAbs spanning the CgA C-terminal domain confirms yet again the assumptions made about the proteolysis of this region. The involvement of the last cleavage sites (400, 409 and 437) is obvious, not only with regard to the concentration measured in pathological plasmas but also in normals for which half the population assessed did not contain any hCgA. Only two samples from carcinoid tumours were highly positive and comparable to the levels obtained with the three other assays. However, this comparison could not allow us to conclude the exact extent to which the proteolysis occurs on the C-terminal domain, nor to establish a relationship with the type of pathology. The degradation is substantially less active upstream in the sequence, as shown by the widespread dispersion obtained with CGS06/CGS04*. There were, nevertheless, a fair number of samples for which the majority of circulating hCgA was apparently truncated from the 373–439 domain, and perhaps from a wider part. The extension of this study to the CGS06/CGS21* or CGS20* systems would be necessary to confirm which of the three cleavage sites (322, 338 or 373) are processed preferentially.

The clinical value of CgA measurement relies on the detection of all its circulating forms. Although it could be speculated that the detection of a more restricted fragment could correlate to total CgA measurement, it has been shown that the assessment of plasma pancreastatin, produced by cleavage at the 243 and 294 dibasic sites, was less discriminative and of less clinical interest than that of total CgA (Stridsberg et al, 1995). Our results show that the hCgA C-terminal end is particularly affected by degradation, but that proteolysis decreases when moving upstream in the sequence. They confirm those of Metz-Boutigue et al (1993) who also found that this region is predominantly processed. Under these circumstances, a mAb against the less affected median part

© Cancer Research Campaign 1999

of hCgA (CGS06) behaves in a comparable manner to a polyclonal antibody, and thus allows the capture of a large number of CgA entities. The association of this mAb with another one having a contiguous epitope (CGS30) also enables the measurement of major fragments and intact hCgA. After these results, this immunoassay is readily included in several experimental protocols, in order to evaluate its clinical value on larger populations as well as on other neuroendocrine pathologies or mixed neoplasia. In a first comparative study, Baudin et al (1998) show that CgA was more sensitive than NSE in a population of patients with gastroenteropancreatic neuroendocrine tumours, MTC and phaeochromocytoma. The results obtained with this immunoradiometric assay also suggest that CgA may be more satisfactory than neuron-specific enolase for the follow-up of such patients.

### ACKNOWLEDGEMENTS

We express our sincere gratitude to P Garcia-Sablone for help in the mapping of monoclonal antibodies and to M Moniatte for mass spectra analysis. We are indebted to Professors E Comoy (Institut Gustave Roussy, Villejuif, France) and D Guilloteau (Hôpital Bretonneau, Tours, France) for kindly providing the pathological plasmas. We are very grateful to B Marshall for reviewing this manuscript and to MH Garnier for her technical assistance.

### REFERENCES

Bader MF and Aunis D (1983) The 97-Kd α-actinin-like protein in chromaffin granule membranes from adrenal medulla: evidence for localization on the cytoplasmic surface and for binding to actin filaments. *Neuroscience* **8**: 165–181

Barbosa JA, Gill BM, Takiyyuddin MA and O’Connor DT (1991) Chromogranin A: post-translational modifications in secretory granules. *Endocrinology* **128**: 174–190

Baudin E, Gigliotti A, Ducreux M, Ropers J, Comoy E, Sabourin JC, Bidart JM, Cailleux AF, Bonacci R, Ruffié P and Schlumberger M (1998) Neuron-specific enolase and chromogranin A as markers of neuroendocrine tumors (in press)

Bender H, Maier A, Wiedenmann B, O’Connor DT, Messner K and Schmidt-Gayk H (1992) Immunoluminometric assay of chromogranin A in serum with commercially available reagents. *Clin Chem* **38**: 2267–2272

Cetin Y and Grube D (1991) Topology of chromogranins in secretory granules of endocrine cells. *Histochemistry* **96**: 301–310

Corti A, Gasparri A, Chen FX, Pelagi M, Brandazza A, Sidoli A and Siccardi AG (1996*a*) Characterisation of circulating chromogranin A in human cancer patients. *Br J Cancer* **73**: 924–932

Corti A, Longhi R, Gasparri A, Chen FX, Pelagi M and Siccardi AG (1996*b*) Antigenic regions of human chromogranin A and their topographic relationships with structural/functional domains. *Eur J Biochem* **235**: 275–280

Curry WJ, Johnston CF, Hutton JC, Arden SD, Rutherford NG, Shaw C and Buchanan KD (1991) The tissue distribution of rat chromogranin A-derived peptides: evidence for differential tissue processing from sequence specific antisera. *Histochemistry* **96**: 531–538

Cussenot O, Villette JM, Valeri A, Cariou G, Desgrandchamps F, Cortesse A, Meria P, Teillac P, Fiet J and Leduc A (1996) Plasma neuroendocrine markers in patients with benign prostatic hyperplasia and prostatic carcinoma. *J Urol* **155**: 1340–1343

Deftos LJ, Nakada S, Burton DW, Di Sant’Agnese PA, Cockett TK and Abrahamsson PA (1996) Immunoassay and immunohistology studies of chromogranin A as a neuroendocrine marker in patients with carcinoma of the prostate. *Urology* **48**: 58–62

Dillen L, De Block J, Van Lear L and De Potter W (1989) Enzyme-linked immunosorbent assay for chromogranin A. *Clin Chem* **35**: 1934–1938

Eriksson B, Arnberg H, Oberg K, Hellman U, Lundqvist G, Wernstedt C and Wilander E (1990) A polyclonal antiserum against chromogranin A and B – a new sensitive marker for neuroendocrine tumours. *Acta Endocrinol* **122**: 145–155

Gill BM, Barbosa JA, Hogue-Angueletti R, Varki N and O’Connor DT (1992) Chromogranin A epitopes: clues from synthetic peptides and peptide mapping. *Neuropeptides* **21**: 105–118

Goumon Y, Strub JM, Moniatte M, Nullans G, Poteur L, Hubert P, Van Dorsselaer A, Aunis D and Metz-Boutigue MH (1996) The C-terminal biphosphorylated proenkephalin-A-(209–237)-peptide from adrenal medullary chromaffin granules possesses antibacterial activity. *Eur J Biochem* **235**: 516–525

Hsiao RJ, Seeger RC, Yu AL and O’Connor DT (1990) Chromogranin A in children with neuroblastoma. *J Clin Invest* **85**: 1555–1559

Hunter WM and Greenwood FC (1962) Preparation of iodine-131 labeled human growth hormone of high specific activity. *Nature* **194**: 495–496

Iacangelo AL and Eiden LE (1995) Chromogranin A: current status as a precursor for bioactive peptides and a granulogenic/sorting factor in the regulated secretory pathway. *Regul Pept* **58**: 65–88

Konecki DS, Benedum UM, Gerdes HH and Huttner WB (1987) The primary structure of human chromogranin A and pancreastatin. *J Biol Chem* **262**: 17026–17030

Metz-Boutigue MH, Garcia-Sablone P, Hogue-Angeletti R and Aunis D (1993) Intracellular and extracellular processing of chromogranin-A. Determination of cleavage sites. *Eur J Biochem* **217**: 247–257

O’Connor DT and Bernstein KN (1984) Radioimmunoassay of chromogranin A in plasma as a measure of exocytotic sympathoadrenal activity in normal subjects and patients with pheochromocytoma. *New Engl J Med* **311**: 764–770

O’Connor DT and Deftos LJ (1986) Secretion of chromogranin A by peptide-producing endocrine neoplasms. *N Engl J Med* **314**: 1145–1151

O’Connor DT, Frigon RP and Sokoloff RL (1984) Human chromogranin A. Purification and characterization from catecholamine storage vesicles of human pheochromocytoma. *Hypertension* **6**: 2–12

O’Farrell PH (1975) High resolution two-dimensional electrophoresis. *J Biol Chem* **72**: 248–254

Simon JP and Aunis D (1989) Biochemistry of the chromogranin A protein family. *Biochem J* **262**: 1–13

Stridsberg M, Oberg K, Li Q, Engstrom U and Lundqvist G (1995) Measurements of chromogranin A, chromogranin B (secretogranin I), chromogranin C (secretogranin II) and pancreastatin in plasma and urine from patients with carcinoid tumours and endocrine pancreatic tumours. *J Endocrinol* **144**: 49–59

Strub JM, Goumon Y, Lugardon K, Capon C, Lopez M, Moniatte M, Van Dorsselaer A, Aunis D and Metz-Boutigue MH (1996) Antibacterial activity of glycosylated and phosphorylated chromogranin A-derived peptide 173–194 from bovine adrenal medullary chromaffin granules. *J Biol Chem* **271**: 28533–28540

Strub JM, Sorokine O, Van Dorsselaer A, Aunis D and Metz-Boutigue MH (1997) Phosphorylation and O-glycosylation sites of bovine chromogranin A from adrenal medullary chromaffin granules and their relationship with biological activities. *J Biol Chem* **272**: 11928–11936

Syversen U, Jacobsen MB, O’Connor DT, Ronning K and Waldum HL (1994) Immunoassays for measurement of chromogranin A and pancreastatin-like immunoreactivity in humans: correspondence in patients with neuroendocrine neoplasia. *Neuropeptides* **26**: 201–206

Taupenot L, Remacle JE, Helle KB, Aunis D and Bader MF (1995). Recombinant human chromogranin A: expression, purification and characterization of the N-terminal derived peptides. *Regul Pept* **56**: 71–88

Watkinson A, Jonsson AC, Davison M, Young J, Lee CM, Moore S and Dockray GJ (1991) Heterogeneity of chromogranin A-derived peptides in bovine gut, pancreas and adrenal medulla. *Biochem J* **276**: 471–479

© Cancer Research Campaign 1999
